# Virulence genes and phylogenetic groups of uropathogenic *Escherichia coli* isolates from patients with urinary tract infection and uninfected control subjects: a case-control study

**DOI:** 10.1186/s12879-021-06036-4

**Published:** 2021-04-17

**Authors:** Seyedeh Elham Rezatofighi, Mahsa Mirzarazi, Mansour Salehi

**Affiliations:** 1 Department of biology, Faculty of Science, Shahid Chamran University of Ahvaz, Ahvaz, 6135743135 Iran; 2 Department of Genetics and Molecular Biology, Medical School, Isfahan University of Medical Sciences, Isfahan, Iran

**Keywords:** Urinary tract infection, Uropathogenic *Escherichia coli*, Virulence factor, Phylogenetic group, Extraintestinal Pathogenic *E. coli*

## Abstract

**Background:**

Urinary Tract Infection (UTI) is one of the most common bacterial infectious diseases which causes considerable morbidity and costly health problems. Uropathogenic *Escherichia coli* (UPEC), the most common pathogen causing UTI, is a highly heterogeneous group of extraintestinal pathogenic *E. coli* (ExPEC) which may carry a variety of virulence factors and belonging to different phylogenetic backgrounds. The current study aimed to investigate the frequency and association between various virulence factors (VFs) and phylogenetic groups of UPEC and commensal isolates.

**Methods:**

UPEC and commensal *E. coli* strains isolated from UTI and feces of healthy humans were compared for the presence of VFs and phylogenetic groups. Association between virulence genes was investigated and cluster analysis was employed.

**Results:**

According to the results, among a 30 virulence markers tested, the pathogenicity-associated island (PAI)*, pap*AH, *papEF*, *fimH, fyuA*, and *traT* genes prevalence were statistically significant in UPEC isolates. A strong association was found between the B2 and D phylogenetic groups and clinical isolates of UPEC; while, commensal isolates were mostly associated with phylogenetic group A. The aggregated VFs scores were more than twice higher in the UPEC isolates in comparison with the commensal isolates. Interestingly, the B2 group in both UPEC and commensal isolates had the highest VF scores. A strong positive association was found between several virulence genes. The clustering results demonstrated that UPEC or commensal *E. coli* isolates were highly heterogeneous due to different composition of their virulence gene pool and pathogenicity islands.

**Conclusion:**

Genetic structure and VFs of UPEC strains vary from region to region; therefore, to control the UTI, the epidemiological aspects and characterization of the UPEC isolates need to be investigated in different regions. Since UPEC isolates are generally originate from the commensal strains, it may be feasible to reduce the UTI burden by interfering the intestinal colonization, particularly in the highly pathogenic clonal lineages such as B2.

## Background

*Escherichia coli* inhabit the large intestine of healthy humans and other warm-blooded animals, but in some instances it can produce a wide range of extraintestinal infections. This bacterium can easily acquire virulence factors (VFs) and mobile genetic elements from related bacteria that leads to different pathogenicity [1]. Extraintestinal pathogenic *E. coli* (ExPEC) isolates are highly complex and have a variety of VFs and may belong to different phylogenetic lineages. These strains cause complicated urinary tract infections (UTIs), bacteremia, and sepsis [2].

UTI is one of the most common infectious diseases accounting for approximately 40% of all nosocomial infections and 10–20% of hospital-acquired infections [3]. UTI is associated with considerable morbidity and costly health problems. They cause a variety of clinical signs from asymptomatic bacteriuria to pyelonephritis, cystitis, and septic shock with multi-organ systems failure [4]. The most common pathogen causing UTI is a heterogeneous group of ExPEC, named uropathogenic *E. coli* (UPEC) [5]. UPEC strains cause 75–95% of uncomplicated and 40–50% of complicated UTIs [3]. Based on the available literature, UPEC strains evolve from non-pathogenic strains by acquiring new VFs through horizontal gene transfer (HGT) [6]. The *E. coli* genome consists of a main core genome and a mobile gene pool that determine pathotype or ecotype specific traits [3]. Various VFs have been attributed to UPEC pathogenesis; however, there is no general agreement regarding the definitive discriminatory virulence factors within this pathotype. UPEC isolates need VFs for colonizing or invading host cells, escaping or disrupting hosts’ immune systems, damaging host tissues, and/or stimulating inflammatory responses. Among variety of VFs, some are generally accepted to be more associated with UPEC [7]. *fim* operon, *pap* operon, and *sfa* genes encode type I fimbriae, P fimbriae and S fimbriae respectively [8, 9]. These structural VFs are the main attachment factors associated with colonization of organism to host cells [7]. Apart from adhesins, some virulence genes encode toxins such as hemolysin (*hly* gene), cytotoxic-necrotizing-factor (*cnf1* gene), and sidrophores (*fyuA* gene) that are mainly involved in intracellular survival, iron-acquisition, escape from immune system, inflammatory response, and host tissue damage [8–10]. UPEC isolates may carry pathogenicity-associated islands (PAIs) which carry sets of different virulence associated genes [8]. Some of these virulence genes are also found in the commensal isolates and are not specific to pathogenic isolates. However, most available studies have only investigated the prevalence of virulence genes in UPEC associated isolates.

Due to variability in the gene content and the possibility of HGT among different *E. coli* isolates, it is vital to understand the genetic basis of differences between commensal and UPEC isolates, to be able to prevent ExPEC and UPEC infections more effectively. This information can be acquired through case-control epidemiological studies [4, 11]. Therefore, in the present study, we investigated the frequency and relationship between different VFs and phylogenetic groups of UPEC and commensal isolates.

## Methods

### Sample collection and analysis

In a case-control study, 702 midstream specimens of urine were collected from patients with an age range from 1 month to 93 years (mean age: 37.07 ± 22.2 years). Sampling was performed randomly. The samples were obtained from patients referred by physicians to medical centers in Isfahan city, Iran, to diagnose urine infection. Urine samples were cultured on a MacConkey (Merck, Germany), Eosin Methylene Blue (Merck, Germany) and blood agar (Merck, Germany) plates. Positive urine cultures with at least 10^5^ cfu/mL and an evaluated white blood cell counts (≥10^4^ leukocyte/mL of urine) were considered UTI positive. UPEC isolates were confirmed using standard biochemical tests including EMB, Methyl Red - Voges-Proskauer (MR-VP), Triple Sugar Iron agar (TSI), and Simmons Citrate agar testing. To confirm the isolates to be *E. coli* and also to evaluate of the quality of extracted DNA, the presence of *uspA* gene was investigated with PCR [11]. This gene encodes the highly specific *E. coli* universal stress protein A. Out of 702 urine specimens, 138 samples were positive for UTI caused by UPEC.

In addition, 30 commensal *E. coli* isolates collected from feces of healthy humans were considered as control. The control samples were collected from volunteers who had no symptoms of disease and not taken antibiotics in the last three months. The study was approved by Ethics Committee of Shahid Chamran University of Ahvaz (Ethics statement No 63/21/8/90). Patients and volunteers were asked to read, accept and sign an informed consent form before any information was collected. Written consent was obtained from the parents for sampling children.

### Virulence genes (VGs)

DNA of UPEC and commensal isolates were extracted using the boiling method. A group of 30 VGs was analyzed. Five separate multiplex PCR were used for the presence of VFs including PAI, *papAH*, *fimH*, *kpsMT III*, *papEF*, *ibeA*, *fyuA*, *bmaE*, *sfa/focDE*, *iutA*, *papG*, *papG* allele I, I^′a^,II, and III, *kpsMT* K1, *hlyA*, *rfc*, *nfaE*, *kpsMT* II, *papC*, *gafD*, *cvaC*, *cdtB*, *focG*, *traT*, *afa/draBC*, *cnf1*, *sfaS*, and *kpsMT* K5. PCR conditions and primers were performed as described by Johnson and Stell [11]. Each positive gene was confirmed with separate PCR. The sum of VFs for each isolate was calculated and regarded as a VF score.

### Phylogenetic analysis

Phylogenetic groups of isolates were investigated using the method of Clermont et al. based on the presence of two genes of *chuA* and *yjaA,* as well as, a DNA fragment TSPE4.C2 [12]. According to the amplification results, the *E. coli* isolates were classified into one of the major phylogenetic groups: A, B1, B2, or D.

### Cluster analysis

Similarity relationships based on composite genomic profiles of the isolates were used to create a dendrogram according to the unweighted pair group method with averaging (UPGMA) supported by the Numerical Taxonomy and Multivariate Analysis System (NTSYS) package version 2.02pc.

### Statistical methods

The sample size was calculated by the Epi Info, a program developed by the Centers for Disease Control and Prevention available via the link: https://www.cdc.gov/epiinfo/index.html. According to a pilot study, Odds Ratio, percent of control exposed, power, alpha, and ratio of controls to cases were considered 5, 12, 80%, 0.05, and 0.3, respectively. Therefore, at least 94 case samples and 29 control samples were required. The Goodman and Kruskal tau coefficient was used to measure the strength of the associations for the cross-tabulation of virulence genes of the *E. coli* isolates from both UPEC and commensal strains. The association between different groups and the presence of the investigated genes was assessed using the Pearson Chi-square test or Fisher’s exact test with SPSS 21.0 software. Moreover, the scores were compared using the Mann–Whitney *U*-test. Results were considered statistically significant at *p* < 0.05.

## Results

### Virulence characteristics

Compared to commensal isolates, the UPEC isolates had a significantly higher prevalence of several VFs including *papAH* and *papEF* (P fimbria), *fimH* (type 1 fimbriae), *fyuA* (siderophore), *traT*, and PAI (*P* < 0.05) (Table 1). Although other genes were also more prevalent in the UPEC isolates than commensal, but their prevalence was not statistically significant (*P* > 0.05) and were considered normal distribution. (Table 1).

**Table 1:**
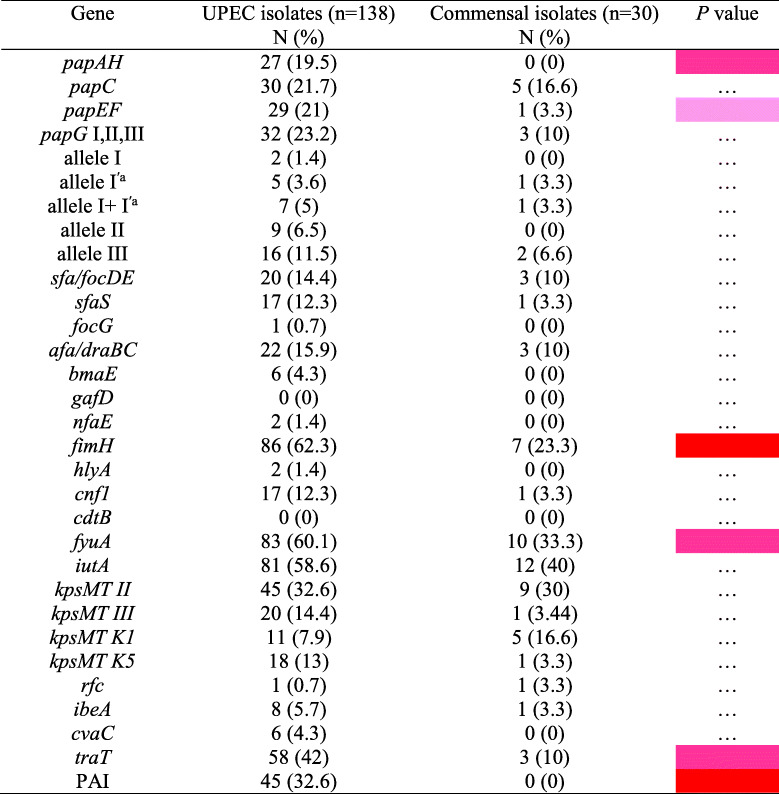
Distribution of virulence-associated traits of uropathogenic and commensal *Escherichia coli* isolates

*P* values (by χ^2^ test or Fisher’s exact test) are shown only if *P* <0.05. The values significantly higher than among the other groups are indicated as follows: *P* < 0.001, *P* < 0.01, *P* < 0.05

### Phylogenetic groups of isolates

The phylogenetic distribution analysis showed a strong association between B2 and D phylogenetic groups and clinical isolates of UPEC; while, commensal isolates were more associated with A phylogenetic group (Table 2). Regarding the strong associations of UTI status with both phylogenetic background and some virulence factors, in the next step, the relationship between phylogenetic groups and virulence factors was investigated.

**Table 2:** Phylogenetic distribution of uropathogenic and commensal *Escherichia coli* isolates

Phylogenetic groups (no of isolates)	Prevalence of phylogenetic groups No (%)		
	UPEC isolates	Commensal isolates	*P* value
	N (%)	N (%)	
A (26)	17 (12.3)	9 (30)	0.02
B1 (16)	16 (11.7)	0 (0)	…
B2 (85)	76 (55)	9 (30)	0.01
D (41)	29 (21)	12 (40)	0.03
Total	138 (100)	30 (100)	

*P* values (by χ^2^ test or Fisher’s exact test) are shown only if P <0.05. UPEC: Uropathogenic *Escherichia coli*

### Phylogenetic distribution of VFs

The distribution of VFs in each phylogenetic group was compared to other phylogenetic groups combined. The results indicated that *papC*, *fimH*, *fyuA*, *iutA*, *kpsMT* II, *kpsMT* K5, and PAI genes were positively associated with group B2, and *papG* allele I^′a^, PAI and *fimH* were also positively associated with group D; while, the *fimH*, *snf1*, and *fyuA* genes were negatively associated with group A, and PAI with group B1, respectively. Other virulence genes had a normal distribution among phylogenetic groups, although group B2 accounted more for most of the virulence genes (Table 3).

**Table 3:**
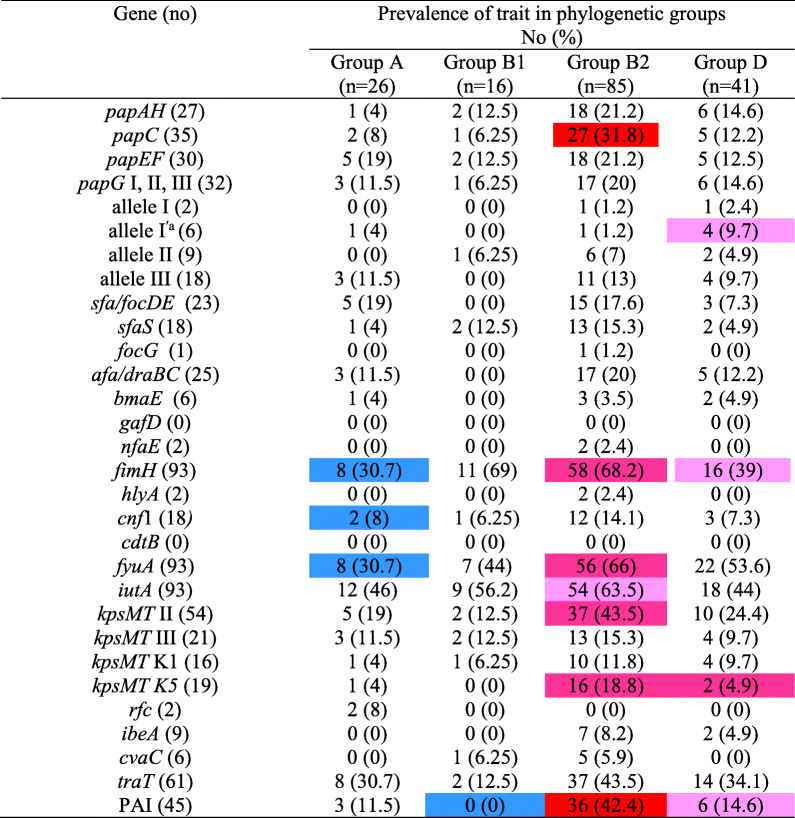
Phylogenetic distribution of virulence-associated traits among uropathogenic and commensal *Escherichia coli* isolates

*P* values were calculated by χ2 test or Fisher’s exact test for comparison of trait of isolates in each group with all other combined groups. The values significantly higher than among the other groups are indicated as follows: *P* < 0.001, *P* < 0.01, *P* < 0.05. The values significantly lower than among the other groups are indicated as follows: *P*< 0.01

### Aggregate VF scores among UPEC and commensal isolates

Aggregate VF scores were calculated by summing the number of the virulence genes present in each commensal or UPEC isolates belonging to each phylogenetic group. Results appeared that aggregate VF scores were more than twice as high among the UPEC isolates (mean: 4.93; range: 2–12) than among the commensal isolates (mean: 2.2; range: 0–8). Different phylogenetic groups also had various aggregate VF scores, so that group B2 had the highest VF score, group D exhibited an intermediate VF score, and scores of the groups A and B1 were significantly lower than other phylogenetic groups. Also, VF scores of all phylogenetic groups of commensal isolates were lower than phylogenetic groups of UPEC isolates (Table 4). Therefore, commensal isolates generally had lower VFs than UPEC isolates.

**Table 4:**
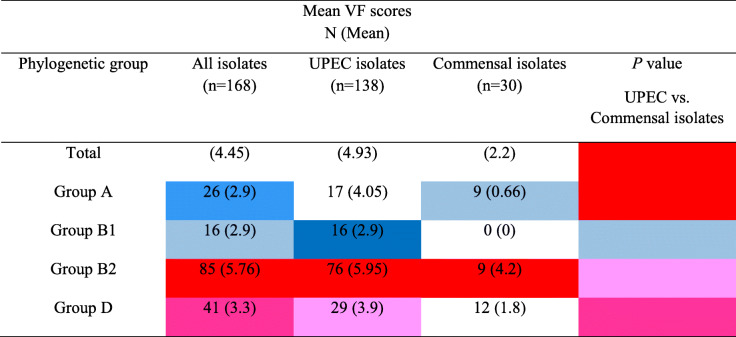
Virulence factor (VF) scores for uropathogenic and commensal *Escherichia coli* isolates, by phylogenetic group

*UPEC* Uropathogenic *Escherichia coli.* The values significantly higher than among the other groups are indicated as follows: *P* < 0.001, *P* < 0.01, *P* < 0.05. The values significantly lower than among the other groups are indicated as follows: *P* < 0.001, *P* < 0.01, *P* < 0.05

### Prevalence of VFs among group B2 of UPEC and commensal isolates

So far, the results showed that the VFs were associated with UPEC isolates and phylogenetic group B2. To explore which origin source of phylogenetic group B2 have more virulence genes, the prevalence of VFs among group B2 of UPEC and commensal isolates were compared. The analysis revealed no large difference between VFs of UPEC isolates over commensal isolates within phylogenetic group B2. Out of 30 VFs only two individual VFs of *traT* and PAI were significantly more prevalent among group B2 of UPEC isolates than group B2 of commensal isolates (Table 5).

**Table 5:** Distribution of virulence-associated traits among uropathogenic and commensal *Escherichia coli* isolates within phylogenetic groups B2

	Prevalence of trait N (%)		
Gene	UPEC isolates (*N*=76)	Commensal isolates (*N*=9)	*P* value
*papAH*	18 (23.7)	0 (0)	…
*papC*	23 (30.3)	4 (44.4)	…
*papEF*	18 (23.7)	0 (0)	…
*papG* I, II, III	17 (22.4)	0 (0)	…
allele I	1 (1.3)	0 (0)	…
allele I^′a^	1 (1.3)	0 (0)	…
allele II	6 (7.9)	0 (0)	…
allele III	10 (13.1)	1 (11.1)	…
*sfa/focDE*	13 (17.1)	2 (22.2)	…
*sfaS*	13 (17.1)	0 (0)	…
*focG*	1 (1.3)	0 (0)	…
*afa/draBC*	16 (21)	1 (11.1)	…
*bmaE*	3 (4)	0 (0)	…
*gafD*	0 (0)	0 (0)	…
*nfaE*	2 (2.6)	0 (0)	…
*fimH*	52 (68.4)	6 (66.6)	…
*hlyA*	2 (2.6)	0 (0)	…
*cnf1*	12 (15.8)	0 (0)	…
*cdtB*	0 (0)	0 (0)	…
*fyuA*	49 (64.5)	7 (77.7)	…
*iutA*	46 (60.5)	8 (88.8)	…
*kpsMT II*	31 (40.8)	6 (66.6)	…
*kpsMT III*	12 (15.8)	1 (11.1)	…
*kpsMT K1*	8 (10.5)	2 (22.2)	…
*kpsMT K5*	15 (19.7)	1 (11.1)	…
*rfc*	0 (0)	0 (0)	…
*ibeA*	7 (9.2)	0 (0)	…
*cvaC*	5 (6.6)	0 (0)	…
*traT*	37 (48.7)	0 (0)	0.004
PAI	36 (47.4)	0 (0)	0.008

*P* values (by χ^2^ test or Fisher’s exact test) are shown only if *P* <0.05. *UPEC* Uropathogenic *Escherichia coli*

### Association between virulence genes

**Fig. 1:**
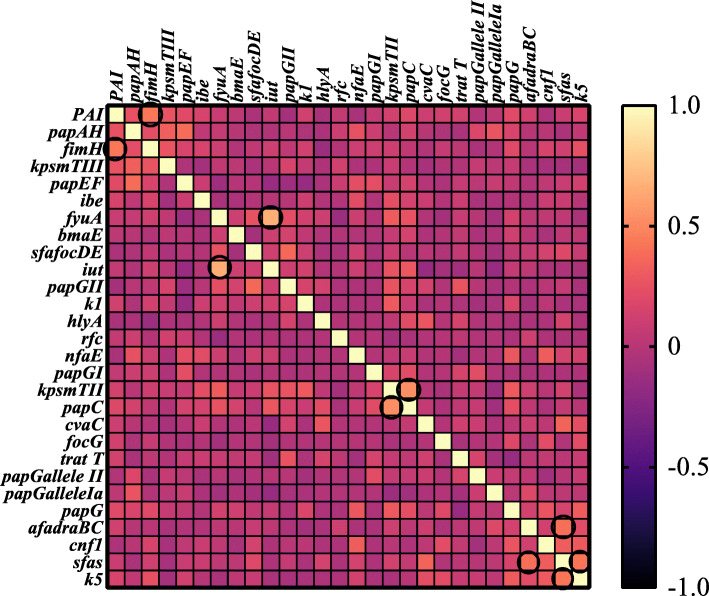
Heat map generated according to statistical association between virulence genes of the *Escherichia coli* isolates derived from uropathogenic and commensal *E. coli* isolates. The strong associations between genes are indicated in the circle. No values were introduced in the case of undetected genes

The association between the various virulence genes of the *E. coli* isolates is shown as a heat map (Fig. 1). A strong positive association was found between the genes of (*fyuA* and *iutA*), (*kpsMT* II and *papC*), (PAI and *fimH*) and (*sfaS* and both genes of *afa/draBC* and *kpsMT* K5). A moderate to weak association was found between the remaining genes.

### Cluster analysis

**Fig. 2:**
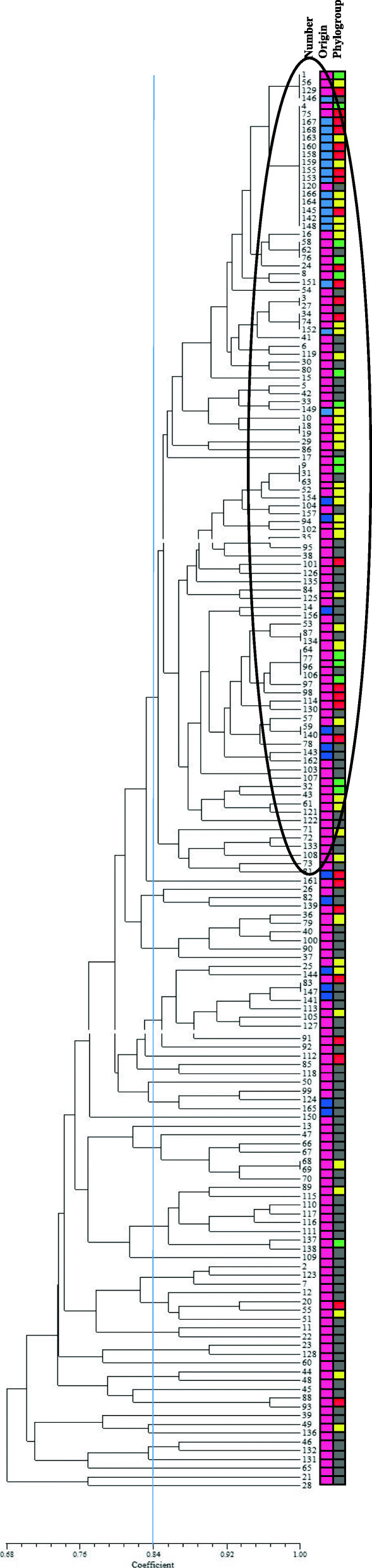
Similarity relationships based on composite genomic profiles and phylogenetic groups of *Escherichia coli* isolates. Commensal and uropathogenic *E. coli* isolate are shown as blue and purple colors respectively. A, B1, B2, and D groups are shown as red, green, gray, and yellow colors respectively. Major cluster is marked on an oval

Cluster analysis using the UPGMA was employed to agglomerate individual isolates into larger clusters and form a dendrogram describing the relationships among UPEC and commensal isolates. Based on the presence or absence of the virulence genes among individual isolates and visualizing genetic associations, one major cluster and several minor clusters were distinguished (Fig. 2). Nearly 80% of commensal isolates were located in the major cluster with the smallest amount of virulence genes. Most of these commensal isolates were correspond to the phylogenetic group A or D. However, some commensal isolates were placed in the other clusters next to the pathogenic isolates with high virulence genes content. These isolates were corresponded to phylogenetic group B2, except for one case. About half of the UPEC isolates were placed in the minor clusters and were related to phylogenetic group B2. These isolates exhibited higher virulence genes content.

## Discussion

To have a better knowledge on the pathogenesis of the UPEC, it is necessary to identify virulence markers of various strains that cause UTIs. VFs, as the potential clinical predictors, help clinicians to manage patients and anticipate the evolution of infection in the host body [6, 13]. However, except for the genetic characteristics of the virulence strains, host factors play an important role in the incidence and outcomes of the infection [14, 15].

Fimbriae and adhesins are frequently reported as VFs in the UPEC isolates [11, 16, 17]. Fimbriae have an important role in establishing and progression of UTI. P-fimbrial adhesins with binding capacity to renal cell receptors cause the specific signaling pathways that trigger mucosal inflammation and tissue damage [18, 19]. Although, in the current study all investigated P fimbriae genes were more prevalent in the UPEC isolates, than the commensal isolates, but the prevalence of *papAH* and *papEF* genes was statistically significant. Correlation association analysis between virulence genes revealed a positive association between *papAH* and *papEF* genes. The *pap* genes are usually chromosomal [2]; therefore, these two genes were probably transmitted together through the chromosome.

Among adhesin genes, *fimH,* the gene that encodes type 1 fimbriae, was common among the UPEC isolates. This VF is attributed to cyctitis-associated UPEC strains and helps to adhere, invade, and form the intracellular bacterial communities (IBCs) [14, 16, 20]. The prevalence of *fimH* was found to be 62.3% that was lower than some studies in Iran [16, 21], but it’s consistent with some previous reports from Mexico, Tunisia, and Iran [6, 22, 23]. In the current study, the *fimH* gene had the most prevalence among virulence genes, which may indicate its critical role in producing UTI. Therefore, FimH could be considered as a potential vaccine candidate. Besides, some studies are investigating this issue. For instance, it has been previously shown that antibodies against FimH can prevent the colonization of UPEC in urinary tract system [24, 25]. The *fimH* gene showed a positive correlation with PAI, indicating the genetic linkages between them.

Although, both siderophore genes of *fyuA* and *iutA* were prevalent in more than 50% of UPEC isolates; the frequency of yersiniabactin (*fyuA* gene) was statistically significant in the UTI producing isolates more than commensal isolates. Moreno et al. also reported a strong association of *fyuA* with urine versus fecal sources [26]. Drawn heat map revealed a strong positive association between two *fyuA* and *iutA* siderophore genes, which indicates the importance of iron absorption systems in pathogenic isolates.

The *traT* was another gene that was statistically significant in UPEC isolates. It expresses a transfer protein that inhibits the classical pathway of complement activation [2]. This gene is a part of *tra* operon of the F-like conjugative plasmids and leads to serum survival [2]. In the other study from Iran, consistent with our results, *traT*, *fyuA*, and *fimH* genes were the most frequently detected VFs in UPEC isolates [27].

ExPEC strains, particularly UPEC isolates, usually contain multiple PAIs with a distinctive combination of VFs; therefore, some isolates may have multiple copies of a VF [13]. In this way, PAIs can play an important role in increasing the pathogenicity of bacteria.

Although, the frequency of other genes was not statistically significant between two groups of UPEC and commensal isolates; but, in the current study, gene expression levels were not investigated. The rate of genes transcription, expression, or the copy number of each gene might be different in these two groups of isolates. Also, to detect virulence genes the PCR method was used, however, due to mutation, some virulence genes may not be accurately detected. Therefore, positive PCR results indicated the presence of genes; but, a negative result does not essentially equivalent to the absence of the corresponding genes, although this phenomenon is scarce [6].

Phylogenetic analysis revealed that the B2 and D (to a lesser extent) were dominant phylogroups of UPEC isolates. The prevalence of VFs was higher among group B2 isolates taken from the urine of patients with acute cystitis than fecal isolates of healthy people [28, 29], which is in line with previous studies conducted in Ethiopia [240], Denmark [30], Pakistan [31], South Korea [18], Poland [32], and Mexico [22]. Therefore, in the producing-UTI isolates, most of the VFs were more frequent than commensal isolates. On the other hand, the UPEC isolates mostly belonged to group B2. Thus, the association between VFs and phylogenetic groups was investigated.

Distribution of VFs in the phylogenetic groups indicated the presence of some genes, including *papC*, *fyuA*, *iutA*,*kpsMT* K5, and *kpsMT*II were positively associated with group B2, *fimH* and PAI with both B2 and D groups, and *papG* allele I^′a^ was associated with group D. Such associations are also reported in other studies [14, 33–35]. Using such evidence scientists can investigate these phylogenetic groups (B2 and D) for VFs, which as-yet is undefined [13].

VF scores of UPEC isolates were higher than commensal isolates. Group B2 in both commensal and UPEC isolates had the highest aggregative VF scores, followed by group D (intermediate), and groups A and B1 (the lowest). Based on the obtained results, it can be concluded that isolates related to the B2 phylogenetic group had a pathogenic potential, regardless of their origin. In this way, these isolates can be a possible candidate for developing a vaccine or drugs.

Co-selection or direct genetic linkage of VFs leads to the common simultaneous appearance of certain VFs [11, 13, 36]. In the current study, the Goodman and Kruskal tau coefficient method and heat map were used to find the strength of the associations of virulence gens. If different studies investigate and identify the associations between genes; it would be feasible to have a better understanding of the role of these genes in how pathogenesis occurs.

UPEC isolates are diverse due to the presence of different virulence genes carried by plasmids, transposons, PAIs, and bacteriophages. These genetic elements may carry antibiotic resistance genes in addition to virulence genes. Plasmids belonging to the IncF incompatibility group were found to encode both VFs and antibiotic resistance genes [37]. However, some researchers believe that multidrug resistant isolates are significantly less virulent than susceptible isolates since antibiotic resistance and virulence do not usually co-evolve simultaneously [38]. Although some studies have found the positive association between virulence traits including iron scavenger receptors with antibiotic resistance [39, 40], there are many contradictions in this regard. For example, in some studies the presence of *hly* gene was associated with sensitivity to fluoroquinolones [41–43] and in other study resistance to fluoroquinolones [8]. Thus, future comprehensive studies are necessary to elucidate the relationship between VFs and antibiotic resistance and the evolutionary direction of bacteria.

The clustering results demonstrated that the UPEC or commensal *E. coli* isolates are genotypically highly heterogeneous. These different patterns of clusters are probably due to chromosomal or plasmid location of virulence genes and vertical (within-lineage) or horizontal (among-lineage) gene transfer phenomena [13]. Additionally, these data emphasize the findings relay on the fact that many UPEC isolates originate from commensal strains without considerable virulence genes content as previously reported in some women, and UTI may cause by a high prevalence of relatively low virulence *E. coli* strains in the fecal reservoir [4]. Also, commensal strains can be potentially pathogenic, when colonizing extra-intestinal tissues. Therefore, by reducing the intestinal colonization of UPEC strains and dealing with *E. coli* virulence mechanisms, the UTIs may be prevented [26]. The epidemiological aspects of the UPEC in different regions need further investigation to find the spread of different isolates and to understand the dissemination of these pathogens to hosts [17].

## Conclusion

In conclusion, in this study, most UPEC isolates were related to group B2, followed by group D. UPEC isolates carried sets of important fimbriae adhesion associated virulence genes especially *papAH*, *papEF*, and *fimH*. The siderophore genes were another noticeable VFs in UPEC isolates and a strong positive association were found between *fyuA* and *iutA* siderophore genes. The results of the present study confirmed that the UPEC or commensal *E. coli* isolates are highly heterogeneous and have complex genetic backgrounds. Therefore, it seems that the epidemiological aspects and characterization of the UPEC isolates needs to be investigated in different regions in different time frames.

## Data Availability

The datasets used and/or analyzed during the current study are available from the corresponding author on reasonable request.

## Abbreviations

VF: Virulence factor
ExPEC: Extraintestinal pathogenic *E. coli*
UTI: Urinary tract infection
PAI: Pathogenicity associated island
UPEC: Uropathogenic *E. coli*
HGT: Horizontal gene transfer
WBC: White blood cell
VG: Virulence gene
UPGMA: Unweighted pair group method with averaging
NTSYS: Numerical Taxonomy and Multivariate Analysis System
IBC: Intracellular bacterial communities
